# Determination of Nitrosamine Drug Substance-Related Impurities Derived from Nortriptyline and Sertraline Using LC-MS/MS: A Comparative Evaluation of Chromatographic Separation and Pharmaceutical Application

**DOI:** 10.3390/ph18111673

**Published:** 2025-11-05

**Authors:** Minki Shim, Ji Yeon Kim, Seungjin Jung, Minkyeong Hong, Sang Beom Han, Dong-Kyu Lee

**Affiliations:** College of Pharmacy, Chung-Ang University, Seoul 06974, Republic of Koreahansb@cau.ac.kr (S.B.H.)

**Keywords:** nortriptyline, sertraline, antidepressant, nitrosamine drug substance related impurities, liquid chromatography-tandem mass spectrometry

## Abstract

**Background/Objectives**: Nitrosamine drug substance-related impurities (NDSRIs) are a class of potent genotoxic impurities that pose a critical risk to patient safety, thereby necessitating the stringent control of pharmaceutical products. Nortriptyline (NORT) and sertraline (SERT) are two widely prescribed antidepressants that form highly potent NDSRIs, *N*-nitroso-nortriptyline (NNORT) and *N*-nitroso-sertraline (NSERT), respectively. Despite these risks, a substantial gap exists in terms of the validated analytical methods for surveillance. Accordingly, this study addressed this need by developing a liquid chromatography–tandem mass spectrometry method for the quantification of NNORT and NSERT in drug products. **Methods**: A comparative evaluation of two reversed-phase columns (general-purpose C18 column and phenyl-hexyl column) was performed to achieve optimal chromatographic resolution of the parent active pharmaceutical ingredients (APIs). **Results**: The phenyl-hexyl column demonstrated superior separation for both NDSRI/API pairs by leveraging π-π interactions to markedly enhance the resolution. This was particularly critical for SERT. The method was fully validated according to the International Council for Harmonization guideline Q2(R1) and demonstrated excellent linearity (r^2^ = 0.998 for both NNORT and NSERT) with limits of quantitation of 20 ng/g for NNORT and 125 ng/g for NSERT. Accuracy was confirmed with recoveries of 96.6–99.4% for NNORT and 98.6–99.4% for NSERT, and precision was acceptable, with relative standard deviation below 3.9% and 1.9%, respectively. The application of this method to commercial products subjected to accelerated stress testing revealed NNORT formation in NORT products with an average concentration of 190 ng/g, as well as NSERT formation in SERT products resulted in an average concentration of 172 ng/g. **Conclusions**: This validated method provides a reliable tool for routine quality control, thereby enabling pharmaceutical manufacturers and regulatory agencies to ensure safety and compliance with widely used antidepressant medications.

## 1. Introduction

Nitrosamine drug substance-related impurities (NDSRIs) have recently emerged as a key class of genotoxic impurities that demand stringent control in pharmaceutical products [1,2,3]. NDSRIs form when a nitroso group replaces a hydrogen atom on a secondary or tertiary amine within the active pharmaceutical ingredient (API) [4,5]. Unlike small-molecule nitrosamines, such as *N*-nitrosodimethylamine, which first raised regulatory concerns in 2018, NDSRIs are unique to each API and form through reactions with residual nitrites found as excipients or contaminants. However, because the formation pathways are not singular, these impurities are often detected even when mitigation strategies, such as nitrite removal, are employed, rendering their complete elimination challenging [6,7]. The difficulty in predicting and controlling their formation prompted the U.S. Food and Drug Administration to issue guidelines in November 2021 that recommended comprehensive risk assessments and confirmatory testing for NDSRIs in all drug products [8].

The potential health risks posed by NDSRIs are evaluated using the Carcinogenic Potency Categorization Approach (CPCA), which often classifies NDSRIs as having equivalent or even greater carcinogenic potential than well-known small-molecule nitrosamines [9,10]. As of February 2025, 115 of the 127 nitrosamine impurities listed were identified as NDSRIs. Notably, 23 NDSRIs are categorized into the high-risk tiers (Classes 1 and 2), which mandate very low acceptable intake (AI) limits, whereas only one small-molecule nitrosamine (*N*-nitroso-N-methylaniline), falls into these categories [11]. Therefore, although the genotoxic potential of an NDSRI is structure-dependent, many can be highly potent, thereby necessitating vigilant monitoring tailored to specific drug substances [12,13].

Among the APIs susceptible to NDSRI formation, antidepressants are of particular concern owing to their widespread and often long-term use, which increases patient exposure to potential impurities [14,15,16]. Nortriptyline (NORT) and sertraline (SERT) are two commonly prescribed antidepressants [17,18] that form class 1 and class 2 NDSRIs, as categorized by the CPCA. An example includes the corresponding impurity *N*-nitroso-nortriptyline (NNORT), which is considered highly potent [19]. Historically, its considerable risk has been underscored by exceptionally low AI limits of 8 –18 ng/day, rendering it a critical target for highly sensitive quantitative analytical methods. More recently, *N*-nitroso-sertraline (NSERT) was reclassified as a class 2 impurity under the CPCA, thus lowering its AI limit to 100 ng/day [20]. These crucial toxicological concerns have prompted regulatory actions, including the recall of NORT products in Canada and Hong Kong in August 2023, thus highlighting the urgent need for robust quality control measures [21,22].

Despite the clear risks, a notable gap exists in the scientific literature in terms of validated analytical methods for these specific NDSRIs. Although numerous multi-analyte methods using gas or liquid chromatography (LC)–mass spectrometry (MS) have been reported for common small-molecule nitrosamines, methods for drug-specific NDSRIs, such as those derived from NORT and SERT, are scarce [23]. To the best of our knowledge, no analytical method for the NNORT has been published. Only one method using a C8 reversed-phase column for the analysis of NSERT has been reported [24]. Previous study developed an LC-MS/MS method for the analysis of NSERT. However, only a single type of column was used without any comparative evaluation of various stationary phases, and the column employed had a relatively large internal diameter, which is not suitable for mass spectrometric analysis. Furthermore, the method was not applied to actual pharmaceutical products. Therefore, to ensure broad applicability in diverse pharmaceutical laboratory settings, comparative evaluation of different stationary phases is required to establish optimal and versatile separation strategies.

Suitably, this study aimed to address this analytical gap by developing and validating a sensitive and selective LC-tandem mass spectrometry (MS/MS) method for the accurate quantification of NNORT and NSERT. To achieve optimal separation from the parent APIs, the chromatographic performance of a general-purpose C18 column was systematically compared with that of a phenyl-hexyl column, which is known for its enhanced selectivity for aromatic compounds. Furthermore, multiple reaction monitoring (MRM) parameters were optimized by characterizing the distinct mass fragmentation patterns of the APIs and their corresponding NDSRIs. A fully validated method was subsequently applied to monitor the presence of these impurities in commercially available NORT and SERT, thereby demonstrating their suitability for routine quality control and regulatory compliance.

## 2. Results and Discussion

### 2.1. Optimization of MS Conditions for API and NDSRI of Two Antidepressant Drugs

**Figure 1:**
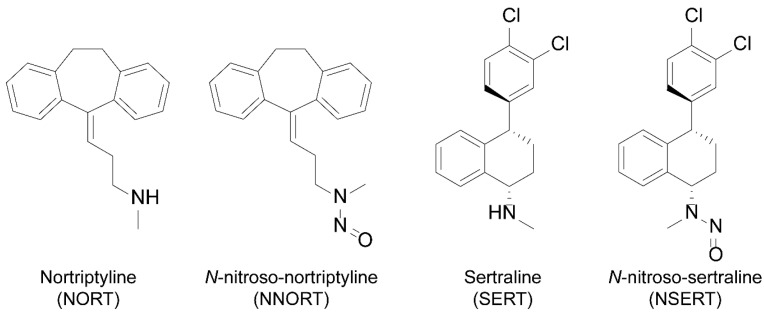
Structures of two APIs (NORT and SERT) and their corresponding NDSRIs (NNORT and NSERT). API, active pharmaceutical ingredient; NDSRIs, nitrosamine drug substance-related impurities.

To develop a robust quantitative method using MS/MS, the fragmentation patterns of NDSRIs of each drug were investigated [25]. The two parent antidepressant drugs, NORT (a tricyclic antidepressant) and SERT (a selective serotonin reuptake inhibitor), possess aromatic structures (Figure 1) that typically lead to complex fragmentation pathways. The relative abundance of the product ions can be highly sensitive to instrumental parameters, including vendor-specific technology, type of collision gas, and mass-to-charge settings. Therefore, empirical optimization within a specific experimental environment is essential.

**Figure 2:**
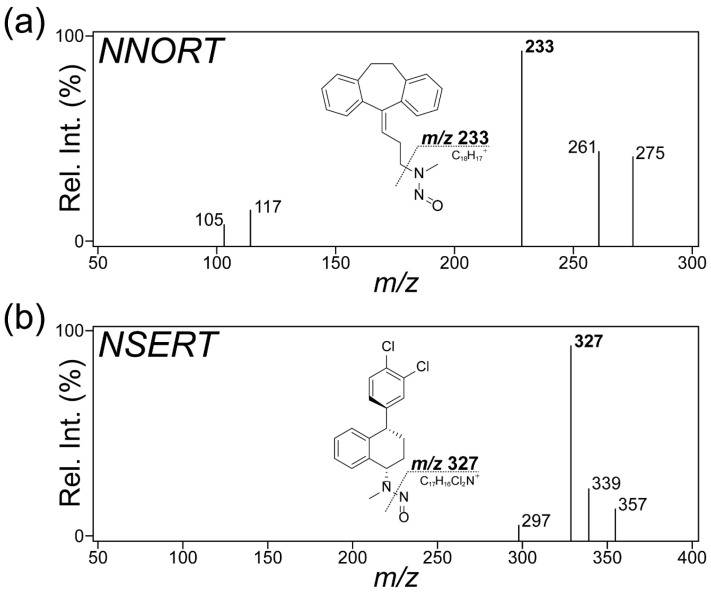
Tandem mass spectrometry (MS/MS) spectra and fragmentation of the quantifier ions. (**a**) *N*-nitroso-nortriptyline (NNORT). (**b**) *N*-nitroso-sertraline (NSERT). Rel. Int., relative intensity.

In the precursor ion scan of NNORT, the dominant species detected was a protonated molecular ion [M + H]^+^ at *m*/*z* 293.2 (Figure 2a). The major fragment ion at *m*/*z* 233 in Figure 2 was generated via cleavage at the point adjacent to the nitroso moiety from protonated NNORT. The transition at *m*/*z* 293–233 was then selected for quantitative transition. Additional daughter ions at *m*/*z* 117 and at *m*/*z* 199 indicated the loss of the nitroso-substituted moiety (Figure S1a,b) [25,26].

The precursor ion for NSERT was primarily observed at *m*/*z* 357.1 as the sodium adduct ion [M + Na]^+^ (Figure 2b). The most common product ion was found at *m*/*z* 327, which arose from nitroso group elimination, and was selected for quantification. Fragmentation patterns that included *m*/*z* 265 and *m*/*z* 241 were selected as qualifier ions (Figure S1c,d). For enhanced chromatographic conditions, MRM transitions at *m*/*z* 293–233 for NNORT and *m*/*z* 357–327 for NSERT were optimized and utilized.

Following the infusion and fragmentation analysis of each NDSRI standard, the product ions that yielded the most intense signal were selected as the quantifier ion, and the second- and third-most abundant signals as the qualifier ions to ensure confident identification.

### 2.2. Comparative Evaluation of Reversed-Phase Stationary Phases for Optimal NDSRI/API Separation

A critical objective was to identify an optimal stationary phase that provides baseline separation of the NDSRI peak from the corresponding, and potentially interfering, API peak. The theoretical XLogP values for NNORT and NORT are 5.0 and 4.5, respectively, whereas those for NSERT and SERT are 5.2 and 4.8, respectively. These high values indicate that all the analytes were highly hydrophobic and were expected to be strongly retained under reversed-phase conditions [27,28].

**Figure 3:**
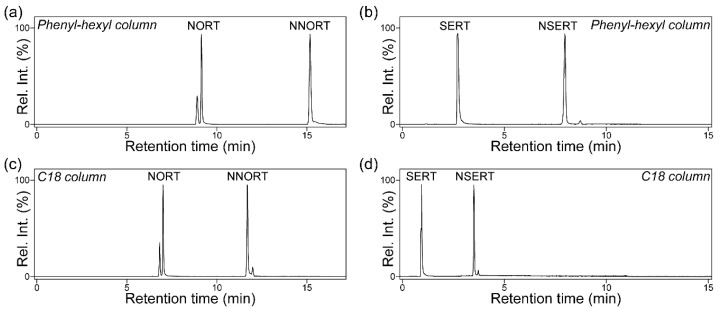
Comparative assessment of C18 and phenyl-hexyl column for separation of active pharmaceutical ingredient (API) and nitrosamine drug substance-related impurities (NDSRI). (**a**) Nortriptyline (NORT) and *N*-nitroso-nortriptyline (NNORT) with a phenyl-hexyl column. (**b**) Sertraline (SERT) and *N*-nitroso-sertraline (NSERT) with a phenyl-hexyl column. (**c**) NORT and NNORT with a C18 column. (**d**) SERT and NSERT with a C18 column. All compounds were analyzed at a concentration of 1 ppm. Rel. Int., relative intensity.

Therefore, the study compared the performance of the following two common reversed-phase columns: a universally applied C18 column and a phenyl-hexyl column, which is known to offer alternative selectivity and improved resolution for aromatic compounds through secondary π-π interactions (Figure 3). The primary analytical challenge in impurity testing is that NDSRIs are typically present at concentrations of less than 1% API. Therefore, when a concentrated sample is required to quantify the NDSRI at its regulatory limit, a massive API peak may become overloaded and lead to pronounced peak broadening. This can cause the API tail to subsume the adjacent NDSRI peak, thereby compromising quantitative accuracy [29]. Consequently, achieving the maximum possible chromatographic resolution between the two compounds is paramount for developing a robust method suitable for routine analysis, even if the separation appears adequate when analyzing simple standard solutions.

The study results demonstrated the superior separation efficiency of the phenyl-hexyl column. Resolution was determined using an equation incorporating the theoretical plate number based on column length and parameters related to chromatographic resolution, since the two columns differed in length (Table S1). The phenyl-hexyl column produced a retention time (RT) gap of 6.0 min for NORT (NDSRI at 15.2 min, API at 9.2 min), with a peak resolution (R_s_) of 44.13 (Figure 3a). The C18 column provided a smaller RT gap of 4.6 min (NDSRI at 11.7 min, API at 7.1 min) and an R_s_ of 30.08 (Figure 3c). The separation improvement for SERT was even more pronounced. The phenyl-hexyl column yielded RTs of 8.0 min (NDSRI) and 2.8 min (API), with an R_s_ of 25.50 (Figure 3b), whereas the C18 column produced RTs of only 3.6 min and 1.1 min and R_s_ of 16.35, respectively (Figure 3d). This resulted in an approximately two-fold greater R_s_ value (44.13 for NNORT, 25.50 for NSERT) on the phenyl-hexyl phase.

Notably, the observed retention behavior did not strictly correlate with hydrophobicity. The theoretical XLogP values for the NDSRI/API pairs were similar (NORT: 5.0 vs. 4.5; SERT: 5.2 vs. 4.8). This suggests that the retention on the phenyl-hexyl column was governed more by hydrophobic retention. The superior resolution, especially for SERT, likely stems from enhanced π-π interactions between the aromatic rings of the analytes and the phenyl groups of the stationary phase. Moreover, the tricyclic structure of NORT may impose steric constraints that hinder the optimal orbital overlap with the stationary phase. In contrast, the structure of SERT may allow a more favorable coplanar orientation of its aromatic rings, thereby promoting stronger interactions and better separation [30].

Although all tested conditions successfully separated the NORT API/NDSRI pair, the phenyl-hexyl column offered a more robust solution. However, the C18 column is inadequate for SERT. Specifically, the minimal separation and short RT of the API render the method highly susceptible to interference from peak broadening and unsuitable for reliable combined API/NDSRI assays.

### 2.3. Method Validation and Assessment of Performance, Applicability and Environmental Compatibility

The analytical method utilizing the optimized phenyl-hexyl column chromatography demonstrated excellent performance across all validation parameters.

Selectivity was confirmed through the analysis of a procedural blank (drug matrix extract), which showed no interfering peaks at the RTs of NNORT and NSERT (Figures S2 and S3).

The method exhibited excellent linearity for both analytes within the matrix (Figure S4a,b), with coefficients of determination values of 0.9987 and 0.9979 for NNORT and NSERT, respectively. This method was also highly sensitive. The limit of quantitation (LOQ) for NNORT was 20 ng/g, which was markedly lower than the AI limit of 54 ng/g. The LOQ for NSERT was 125 ng/g, which enabled quantification at levels four times lower than its AI limit of 500 ng/g [31]. The LOQ difference of around sixfold between NNORT and NSERT is likely due to the two compounds’ distinct chemical structures. NNORT, with its tertiary aryl/alkyl amine structure and high electron density, is more easily ionized in positive ESI mode. In contrast, the presence of electron-withdrawing substituents in NSERT lowers the basicity of its amine group, resulting in reduced protonation and lower ionization efficiency. The LOQ of NSERT 125 ng/g is remarkably lower than that reported in the previous study (5000 ng/g), which is likely due to the use of different chromatographic columns and a more advanced mass spectrometry instrument in our study [24].

The accuracy and precision were outstanding at low, medium, and high concentrations (Table 1). The intra-day accuracy (recovery) for NNORT ranged from 96.0% to 99.3%, with precision (relative standard deviation [RSD]) values between 0.52% and 1.81%. Inter-day analysis over three days yielded similarly high recoveries (96.6% to 99.4%) and excellent precision (RSD ≤ 1.40%), thereby confirming the consistency of the method over time. The intra-day results for NSERT showed recoveries of 99.3 ± 0.23% (low), 99.4 ± 1.87% (medium), and 98.6 ± 1.19% (high), with RSDs of 0.2%, 1.6%, and 3.1%, respectively. Inter-day validation was also robust, with recoveries of 98.6–99.3% and RSDs of 0.4–1.0%.

Robustness was evaluated by assessing the impact of minor deliberate changes in the flow rate on the analyte RT, which showed no substantial deviations (Table 2). Specifically, the flow rate was altered by ±0.05 mL/min and the column temperature was adjusted by ±4 °C to verify the stability of the method. The RT RSDs for NNORT ranged from 0.05% to 0.08%, whereas the RSD of the standard-to-IS (STD/IS) peak area ratio ranged from 0.06% to 4.21%. The tailing factor (TF) was between 1.46 and 1.62, and the R_s_ ranged from 2.58 to 2.87. The RT RSDs, STD/IS ratio, RSDs, TF, and R_s_ values ranged from 0.16% to 0.44%, 1.61% to 5.75%, 0.98 to 1.09, and 5.70 to 12.15, respectively. These variations in chromatographic parameters did not adversely affect method performance, thereby demonstrating that the developed methods provide sufficient robustness for reliable quantification of both NNORT and NSERT.

The verified analytical method of NSERT demonstrated slightly better greenness, practicality and analytical performance than the previously reported method [24]. The environmental and sustainability profiles, as well as the applicability, of the analytical method were evaluated using automated assessment tools, including analytical greenness calculator metric (AGREE), blue applicability grade index (BAGI), and red analytical performance index (RAPI). The AGREE score was 0.42, slightly higher than that of the reference (0.41), likely due to the lower flow rate and reduced solvent consumption during sample preparation (Table S2). In the BAGI evaluation, the developed method achieved a score of 65.0, while the reference method obtained a score of 60.0, reflecting the smaller injection volume used (15 µL versus 50 µL). The RAPI evaluation yielded scores of 70.0 and 62.5 for the verified and reference methods, respectively, since the higher score for the validated method was obtained by evaluating reproducibility over a lower concentration range. These findings suggest that the verified NSERT analytical method provides better environmental compatibility and analytical reliability than the previous study.

### 2.4. Monitoring of NNORT and NSERT in Their Respective Drug Products

The validated method was applied to screen for NDSRIs in finished NORT and SERT products currently marketed in South Korea (Figure S5). Considering that only one product is currently marketed in South Korea, three different batches of a single product were analyzed for NORT. In contrast, products from six manufacturers with a high market share were procured for SERT.

As anticipated, owing to stringent regulatory oversight, no NDSRIs were detected in most recently marketed products because their impurity content was rigorously investigated prior to commercial release. To simulate potential NDSRI formation during the product shelf life, long-term storage at room temperature was conducted for one year.

Following the long-term period, the three batches of the NORT product showed pronounced NNORT formation, with detected levels of 203.0 ± 1.2, 188.3 ± 19.1, and 180.9 ± 28.9 ng/g, respectively, all of which exceeded the maximum permissible limit of 54 ng/g (Table 3). In contrast, the six SERT products showed minimal NSERT formation, even after long-term storage testing. Levels were predominantly below the LOQ, with quantifiable amounts in the range of 50.4 ± 5.4 to 469.4 ± 91.7 ng/g, thus demonstrating a lower propensity for NDSRI formation under these conditions.

## 3. Materials and Methods

### 3.1. Chemicals and Reagents

NNORT-d_3_, NNORT, NSERT-^13^C, ^15^N, and d_3_ were purchased from Toronto Research Chemicals (Toronto, ON, Canada). NSERT, NORT and SERT were obtained from TLC Pharmaceutical Standards Ltd. (Newmarket, ON, Canada). Water and methanol (HPLC grade) were obtained from J.T. Baker (Phillipsburg, NJ, USA). Formic acid (MS grade) was purchased from Sigma-Aldrich (St. Louis, MO, USA). NORT and SERT drug products were purchased from a local pharmaceutical manufacturer.

### 3.2. Sample Preparation

The NORT and SERT standard solutions, and their corresponding *N*-nitroso derivatives (NNORT and NSERT) were prepared in methanol at a concentration of 1 mg mL^−1^ and serially diluted with 0.1% formic acid in 95:5 (*v*/*v*) water: methanol. Final concentrations for calibration were 20, 200, 1000, 2000, and 4000 ng g^−1^ for NNORT, and 125, 250, 375, 500, and 600 ng g^−1^ for NSERT. Internal standard solutions which contained 2.7 ng mL^−1^ NNORT-d_3_ and 10 ng mL^−1^ NSERT-^13^C, ^15^N, and d_3_ were prepared similarly.

Sample preparation for the drug substance was performed as follows. First, NORT and SERT were prepared by finely pulverizing more than 20 tablets of each formulation using a mortar and pestle. Thereafter, NORT (150 mg) and SERT (200 mg) were accurately weighed and transferred from the resulting homogeneous powders into a 50-mL centrifuge tube. Subsequently, 3 mL of 0.1% formic acid in 95:5 (*v*:*v*) water: methanol was added for NORT, and 5 mL of 0.1% formic acid in 40:60 (*v*:*v*) water: methanol was added for SERT. The mixture was vortexed for 30 s, ultrasonicated for 30 min, and centrifuged at 3500× *g* for 30 min. An aliquot of 1 mL of supernatant was transferred into a 1.5-mL microcentrifuge tube and centrifuged at 13,000× *g* for 20 min, and the supernatant was filtered through a 0.22-μm polytetrafluoroethylene filter. Subsequently, 100 μL of the filtrate was transferred into a 2 mL vial containing an insert for LC-MS/MS analysis.

### 3.3. LC-MS/MS Analysis

#### 3.3.1. LC-MS/MS Analysis of NORT

The NNORT and NORT samples were analyzed using an ACQUITY UPLC I-Class PLUS System (Waters Corporation, Milford, MA, USA) coupled with a Xevo TQ tandem quadrupole mass spectrometer (Waters). The following two columns were tested for analyte separation and maintained at 40 °C in the column oven: Kinetex C18 column (2.1 × 100 mm i.d., 2.6 μm, Phenomenex, Torrance, CA, USA) and Kinetex phenyl-hexyl column (3.0 × 150 mm i.d., 2.6 μm, Phenomenex, Torrance, CA, USA). Detailed analytical conditions are provided in Table S3. Analytes were monitored at ion transitions of *m*/*z* 293.2 → 233.2 in the MRM mode under positive ionization using an electrospray ionization (ESI) source (Table 4).

#### 3.3.2. LC-MS/MS Analysis of SERT

A 1290 Infinity II LC System (Agilent Technologies, Santa Clara, CA, USA) equipped with a 6495D triple quadrupole (Agilent Technologies) was used for NSERT and SERT sample analysis. Similarly to NORT analysis, chromatographic separation was performed with both a Kinetex phenyl-hexyl column (3.0 × 150 mm i.d., 2.6 μm, Phenomenex, Torrance, CA, USA) and a Kinetex C18 column (2.1 × 100 mm i.d., 2.6 μm, Phenomenex, Torrance, CA, USA). Further details on analytical parameters are presented in Table S4. In the MRM mode with positive ionization, analytes were quantified by monitoring the ion transition of *m*/*z* 357.1 → 326.9 using an ESI source (Table 4).

### 3.4. Method Validation

The developed HPLC method was validated in accordance with the ICH Guideline Q2(R1), “Validation of Analytical Procedures.” The validation process assessed selectivity, linearity, range, limit of detection (LOD), LOQ, accuracy, precision, and robustness [32]. Selectivity was established by comparing the chromatograms of the standard spiked in the neat solvent, procedural blank, and matrix-matched standard, which contained all formulation excipients without the API. The resulting chromatograms were monitored for interfering signals at specific RTs for each target analyte.

The LOQ was defined as the lowest concentration on the calibration curve (CS1) established at a signal-to-noise (S/N) ratio of approximately 10:1. The LOD was determined by identifying the concentration yielding an S/N ratio of approximately 3:1. The linearity of the method was determined by constructing a five-point calibration curve, with each concentration analyzed in triplicate. The reportable range was established from the LOQ to 120% of the specified limit for each impurity. These specification limits were calculated based on the acceptable daily intakes, corresponding to 54 ng/g for NORT and 500 ng/g for SERT, with the highest calibration point (CS5) set at 120% (Table 5).

Accuracy and precision were evaluated at three concentration levels across the analytical range: low (LOQ and CS1), medium (CS3), and high (CS5). For each level, samples were prepared and analyzed in triplicate. Accuracy was expressed as the mean percent recovery, whereas precision was reported as the RSD (%). To assess repeatability (intra-day precision) and intermediate precision (inter-day precision), analyses were conducted on the same day and on three separate days.

The robustness of the analytical method was assessed by introducing minor variations to the established chromatographic conditions. The column temperature and mobile phase flow rate were intentionally altered, and the effects of these changes on the analyte RTs were monitored to confirm the reliability of the method under various conditions.

## 4. Conclusions

In this study, a highly sensitive and selective LC-MS/MS method was developed and validated for the determination of two critical NDSRIs (NNORT and NSERT) using their respective APIs (NORT and SERT). A key finding was the superior performance of the phenyl-hexyl stationary phase compared to that of a conventional C18 column. The C18 phase was inadequate for reliable separation of NSERT from its parent API, rendering the phenyl-hexyl column essential for a robust combined assay. Accurate quantification requires a high chromatographic resolution to overcome the challenge of potential peak overload from the concentrated API. Accordingly, the phenyl-hexyl column effectively addressed this need by leveraging its unique π-π interactions, yielding approximately 1.5-fold improvement in resolution for both NORT (44.13) and SERT (25.50) compared to that of a standard C18 column (30.08 and 16.35, respectively) under identical analytical conditions. The method was rigorously validated in accordance with the ICH guidelines and proved to be linear, accurate, precise, and robust for its intended purpose. Furthermore, unlike the previous study, which focused on method validation, the applicability of the method evaluated using actual pharmaceutical products in this study, and the method effectively monitored NDSRI levels in commercial drug products [24]. Moreover, accelerated stress testing highlighted a greater propensity for NNORT formation in NORT products compared to NSERT formation in SERT products under the tested conditions. Ultimately, this study provides the pharmaceutical industry and regulatory bodies a robust and essential analytical tool to monitor and control potent genotoxic impurities, thereby improving the safety and quality of essential antidepressant medications. However, the present study compared chromatographic separation using columns with different lengths and internal diameters, which limited the accuracy of the resolution comparison. Additionally, the monitoring experiment involved a relatively large quantity of 20 tablets. Future studies should compare columns with identical specifications but different stationary phases and minimize the number of tablets used in accordance with pharmacopeial and regulatory guidelines to enhance the greenness of the analytical procedure.

## Figures and Tables

**Table 1:** Analytical method validation results for NNORT and NSERT.

Compound	Retention Time (min)	Conc. Range (ng/g)	Calibration Curve	R^2^	LOD (ng/g)	LOQ (ng/g)	Spiked Conc. (ng/g)	Intra-Day		Inter-Day	
								Recovery (%)	CV (%)	Recovery (%)	CV (%)
NNORT	15.19	20–4000	Y = 1.641x − 0.07364	0.9984	6.7	20	20	99.4 ± 3.82%	3.84%	97.4 ± 3.86%	3.96%
							1000	96.6 ± 0.72%	0.74%	97.1 ± 2.08%	2.14%
							4000	99.4 ± 1.76%	1.77%	99.1 ± 0.78%	0.79%
NSERT	7.95	125–600	Y = 0.00249x − 0.0291	0.9979	42.5	125	125	99.3 ± 0.23%	0.23%	99.2 ± 0.42%	0.43%
							375	99.4 ± 1.87%	1.88%	98.6 ± 0.53%	0.54%
							600	98.6 ± 1.19%	1.21%	99.3 ± 0.44%	0.44%

NNORT, *N*-nitroso-nortriptyline; NSERT, *N*-nitroso-sertraline; Conc., concentration; R^2^, coefficients of determination; LOD, limit of detection; LOQ, limit of quantitation; CV, coefficient of variation.

**Table 2:** Robustness verification of NNORT and NSERT quantification method.

	NNORT				NSERT			
	RT RSD	STD/IS Ratio RSD	TF (±STDEV)	R_s_ (±STDEV)	RT RSD	STD/IS Ratio RSD	TF (±STDEV)	R_s_ (±STDEV)
Column temperature −4 °C	0.05%	2.32%	1.46 (±0.34)	2.67 (±0.16)	0.38%	1.61%	0.98 (±0.07)	5.70 (±2.31)
Column temperature +4 °C	0.05%	3.00%	1.55 (±0.14)	2.78 (±0.11)	0.44%	2.50%	1.09 (±0.06)	6.01 (±0.70)
Flow rate −0.05 mL/min	0.06%	0.06%	1.62 (±0.18)	2.58 (±0.06)	0.16%	2.03%	0.99 (±0.05)	10.89 (±2.55)
Flow rate +0.05 mL/min	0.08%	4.21%	1.46 (±0.46)	2.87 (±0.11)	0.16%	5.75%	1.01 (±0.08)	12.15 (±1.17)

NNORT, *N*-nitroso-nortriptyline; NSERT, *N*-nitroso-sertraline; RSD, relative standard deviation; TF, tailing factor; R_s_, resolution; STDEV, standard deviation; STD/IS, standard-to-IS.

**Table 3:** Quantification of NNORT and NSERT in the respective drug products (NNORT AI: 54 ng/g; NSERT AI: 500 ng/g).

NNORT Concentration (ng/g)		NSERT Concentration (ng/g)	
Batch_01	203.0 ± 1.2	Product_01	84.8 ± 13.6
Batch_02	188.3 ± 19.1	Product_02	196.5 ± 15.1
Batch_03	180.9 ± 28.9	Product_03	469.4 ± 91.7
		Product_04	54.7 ± 8.6
		Product_05	176.4 ± 27.0
		Product_06	50.4 ± 5.4

NNORT, *N*-nitroso-nortriptyline; NSERT, *N*-nitroso-sertraline; AI, acceptable intake.

**Table 4:** MRM parameters of NNORT and NSERT and isotope-labeled internal standards.

Compound	Precursor Ion (*m*/*z*)	Product Ion (*m*/*z*)	Collision Energy (eV)
NNORT	293.2	**233.2 ^1^**	10
	293.2	117.2	20
	293.2	199.2	30
NNORT-d_3_	296.2	**233.2 ^1^**	10
	296.2	105.2	30
NSERT	357.1	**326.9 ^1^**	8
	357.1	265.0	23
	357.1	241.0	30
NSERT-^13^C,^15^N,d_3_	362.0	**332.0 ^1^**	8
	362.0	280.0	23

^1^ Bold and underlined values indicate quantitative ion for each compound. MRM, multiple reaction monitoring; NNORT, *N*-nitroso-nortriptyline; NSERT, *N*-nitroso-sertraline.

**Table 5:** Calibration range of NNORT and NSERT.

Calibration Point	Concentration (ng/g)	
	NNORT	NSERT
CS1	20	125
CS2	200	250
CS3	1000	375
CS4	2000	500
CS5	4000	600

NNORT, *N*-nitroso-nortriptyline; NSERT, *N*-nitroso-sertraline.

## Data Availability

The original contributions presented in this study are included in the article/Supplementary Materials. Further inquiries can be directed to the corresponding author.

## Supplementary Materials

The following supporting information can be downloaded at: https://www.mdpi.com/article/10.3390/ph18111673/s1. Table S1: Retention, separation and efficiency parameters of analytical methods for NNORT and NSERT; Table S2: Analytical parameters and validation results of this study and previous study; Table S3: Analytical conditions for NNORT analysis; Table S4: Analytical conditions for NSERT analysis; Figure S1: MS/MS spectra illustrating the fragmentation of the qualifier ions for NNORT and NSERT; Figure S2: Selectivity confirmation via comparison of chromatograms; Figure S3: Selectivity confirmation via comparison of chromatograms; Figure S4: Verification of linearity over a range including AI concentrations. Calibration curve of (a) NNORT and (b) NSERT; Figure S5: Chromatograms of NNORT (a) and NSERT (b) in commercial drug products.

## Abbreviations

The following abbreviations are used in this manuscript:

NDSRIs	nitrosamine drug substance-related impurities
NORT	nortriptyline
SERT	sertraline
NNORT	*N*-nitroso-nortriptyline
NSERT	*N*-nitroso-sertraline
LC	liquid chromatography
MS	mass spectrometry
MS/MS	tandem mass spectrometry
API	active pharmaceutical ingredient
CPCA	Carcinogenic Potency Categorization Approach
AI	acceptable intake
MRM	multiple reaction monitoring
RT	retention time
R_s_	peak resolution
LOQ	limit of quantitation
RSD	relative standard deviation
STD/IS	standard-to-IS
TF	tailing factor
ESI	electrospray ionization
LOD	limit of detection
HPLC	high-performance LC
S/N	signal-to-noise
