# Supplementary material for: Determination of Nitrosamine Drug Substance-Related Impurities Derived from Nortriptyline and Sertraline Using LC-MS/MS: A Comparative Evaluation of Chromatographic Separation and Pharmaceutical Application
Source: Pharmaceuticals (Basel). 2025 Nov 5;18(11):1673. doi: 10.3390/ph18111673 (PMC12655250; doi:10.3390/ph18111673)
Supplement: Supplementary file 1 [file pharmaceuticals-18-01673-s001.zip › pharmaceuticals-3949832-supplementary.pdf]

Supplementary Material

Table S1. Retention, separation and efficiency parameters of analytical methods for NNORT and NSERT

Column	Target	Retention		Separation		Efficiency
		t_r	k	R_s	α	*N*
C18	NNORT	11.7	18.5	30.08	1.7	93,626
	NSERT	3.6	5.0	16.35	6.0	8,864
Phenyl-hexyl	NNORT	15.2	7.4	44.13	1.8	199,994
	NSERT	8.0	3.4	25.50	6.2	24,622

NNORT, N-nitroso-nortriptyline; NSERT, N-nitroso-sertraline; t_r, retention time; k, retention factor; R_s, resolution; α, selectivity factor; N, number of theoretical plates.

Table S2. Analytical parameters and validation results of this study and previous study

Parameter	This study	Chagarlamudi et al. [24]
**Instruments**	Agilent 1290 Infinity II LC System with 6495D triple quadrupole LC–MS/MS system	Shimadzu LCMS-8060NX triple quadrupole LC–MS/MS system
**Column**	Kinetex Phenyl-hexyl (3.0x150 mm, 2.6 μm)	Waters Xbridge C8 (4.6×150 mm, 3.5 μm)
**Mobile phase**	Phase A: 0.1% formic acid in water<br>Phase B: 0.1% formic acid in methanol	Phase A: 0.1% formic acid in water<br>Phase B: 0.1% formic acid in methanol
**Flow rate**	0.4 mL/min	0.8 mL/min
**Injection volume**	15 μL	50 μL
**Column temperature**	40 ℃	40 ℃
**LOD**	42.5 ng/g	3000 ng/g
**LOQ**	125 ng/g	5000 ng/g
**linear range**	125-600 ng/g	5000–75000 ng/g

LOD, limits of detection; LOQ, limits of quantitation.

Table S3. Analytical conditions for NNORT analysis

Instrument	Parameter	Conditions		
**LC**	Column	Kinetex C18 (2.1x100 mm, 2.6 μm)		
		Kinetex Phenyl-hexyl (3.0x150 mm, 2.6 μm)		
	Mobile phase	Phase A: 0.1% formic acid in water		
		Phase B: 0.1% formic acid in methanol		
	Flow rate	0.4 mL/min		
	Injection volume	20 μL		
	Column temperature	40 ℃		
	Gradient	Time (min)	A (%)	B (%)
		0	95	5
		3	95	5
		5	90	10
		10	40	60
		13	20	80
		13.1	5	95
		16	5	95
		16.1	95	5
		18	95	5
**MS/MS**	Capillary voltage	3.0 kV		
	Cone voltage	25 V		
	Desolvation gas temperature	250 ℃		
	Desolvation gas flow	500 L/h		
	Cone gas flow	150 L/h		
	Nebulizer pressure	7.0 bar		

NNORT, N-nitroso-nortriptyline; LC, liquid chromatography; MS/MS, tandem mass spectrometry.

Table S4. Analytical conditions for NSERT analysis

Instrument	Parameter	Conditions		
**LC**	Column	Kinetex C18 (2.1x100 mm, 2.6 μm)		
		Kinetex Phenyl-hexyl (3.0x150 mm, 2.6 μm)		
	Mobile phase	Phase A: 0.1% formic acid in water		
		Phase B: 0.1% formic acid in methanol		
	Flow rate	0.4 mL/min		
	Injection volume	15 μL		
	Column temperature	40 ℃		
	Gradient	Time (min)	A (%)	B (%)
		0	40	60
		3	20	80
		10	20	80
		10.1	40	60
		15	40	60
**MS/MS**	Capillary voltage	3300 V		
	Nozzle voltage	1200 V		
	Drying gas temperature	230 ℃		
	Drying gas flow	13 mL/min		
	Sheath gas temperature	370 ℃		
	Sheath gas flow	11.5 mL/min		
	Nebulizer pressure	33 psi		

NSERT, N-nitroso-sertraline; LC, liquid chromatography; MS/MS, tandem mass spectrometry.

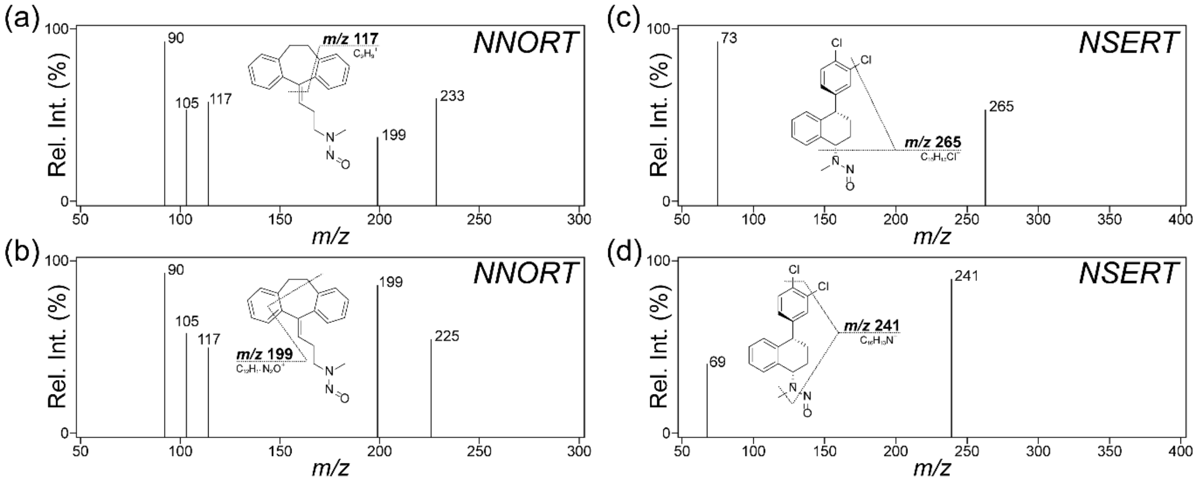

Figure S1. Tandem mass spectrometry (MS/MS) spectra illustrating the fragmentation of the qualifier ions for *N*-nitroso-nortriptyline (NNORT) and , *N*-nitroso-sertraline (NSERT). Spectra for NNORT were acquired at collision energies of (a) 20 eV and (b) 30 eV. Spectra for NSERT were acquired at (c) 20 eV and (d) 30 eV.

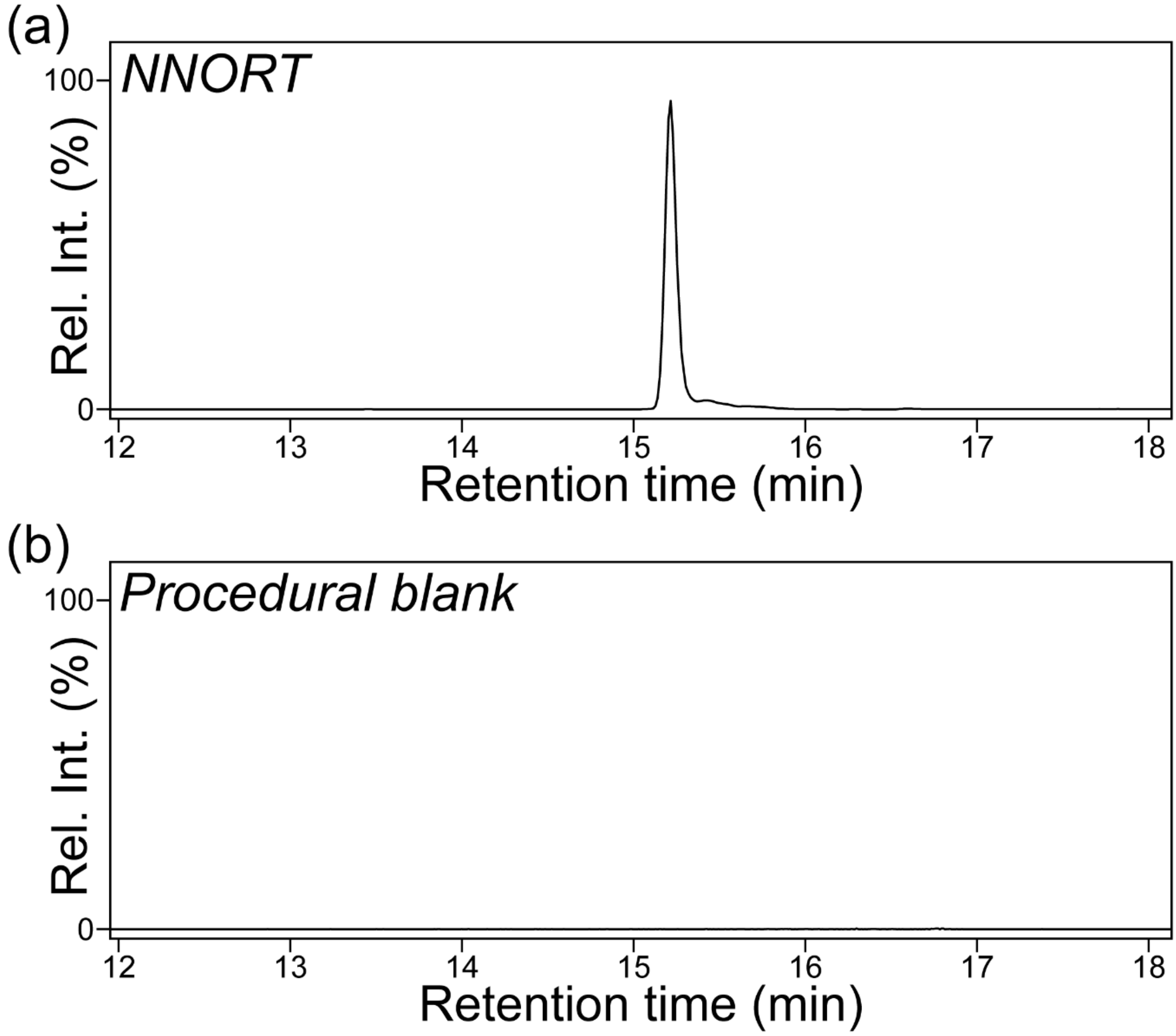

Figure S2. Selectivity confirmation via comparison of chromatograms. for (a) *N*-nitroso-nortriptyline (NNORT). (b) Procedural blank. Rel. Int., relative intensity.

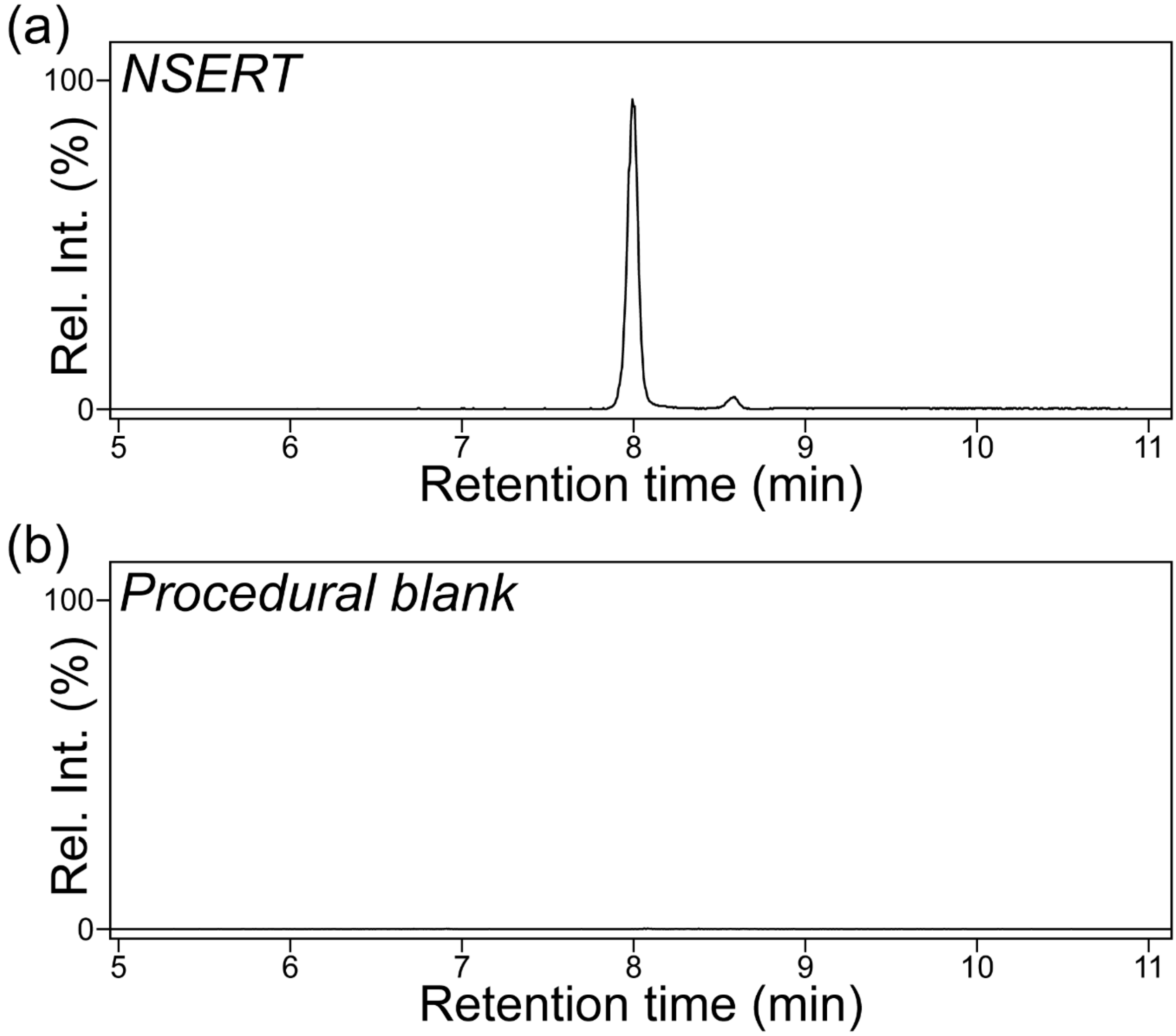

Figure S3. Selectivity confirmation via comparison of chromatograms. (a) *N*-nitroso-sertraline (NSERT). (b) Procedural blank. Rel. Int., relative intensity.

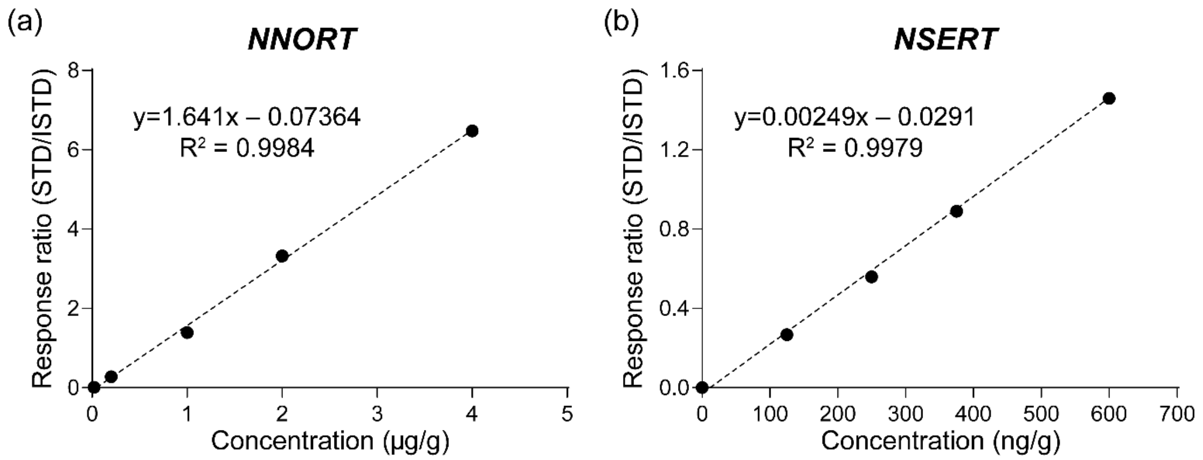

Figure S4. Verification of linearity over a range including acceptable intake (AI) concentrations. Calibration curve of (a) N-nitroso-nortriptyline (NNORT) and (b) N-nitroso-sertraline (NSERT).

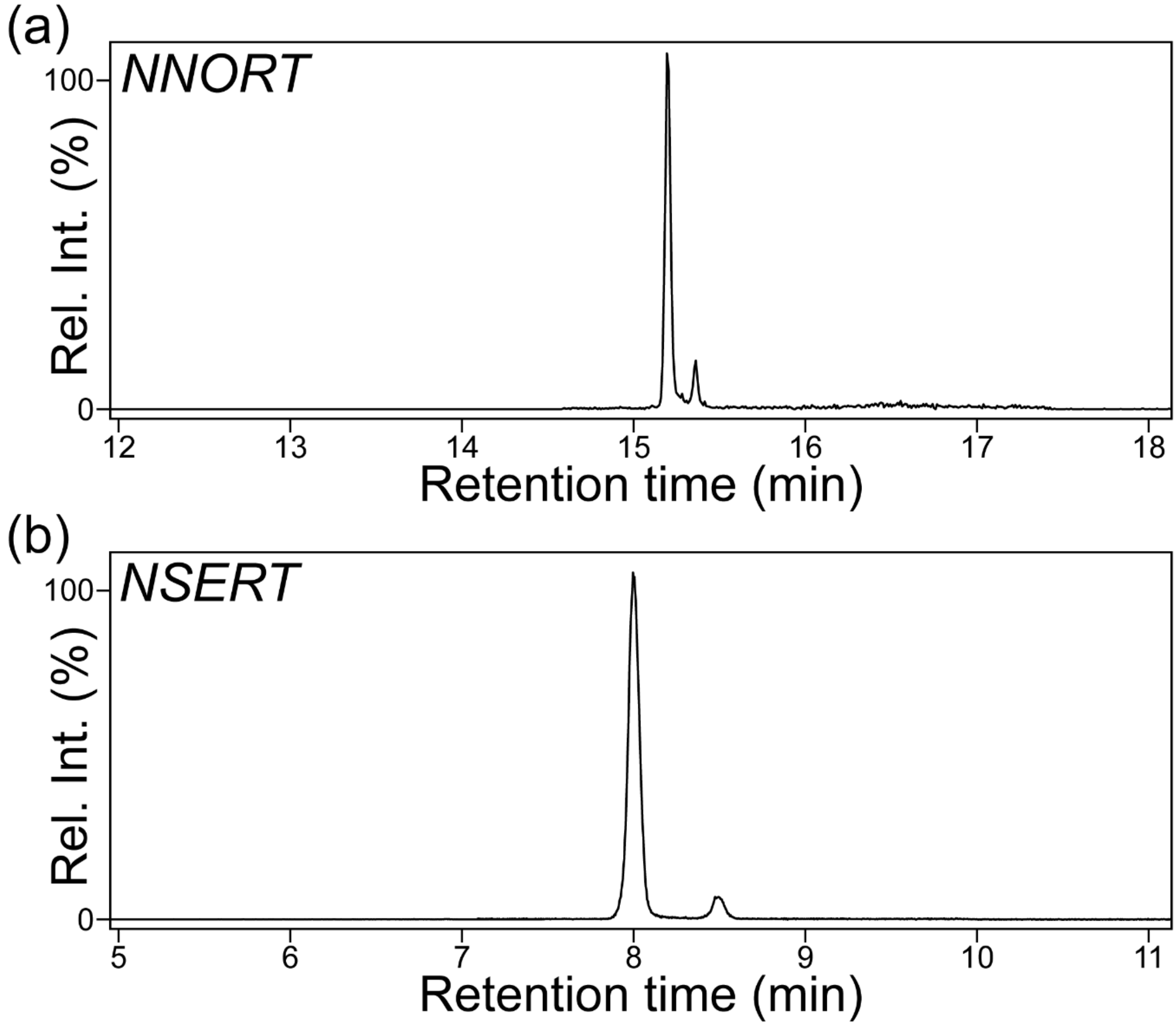

Figure S5. Chromatograms of NNORT (a) and NSERT (b) in commercial drug products.
